# Connectivity, Pharmacology, and Computation: Toward a Mechanistic Understanding of Neural System Dysfunction in Schizophrenia

**DOI:** 10.3389/fpsyt.2013.00169

**Published:** 2013-12-24

**Authors:** Alan Anticevic, Michael W. Cole, Grega Repovs, Aleksandar Savic, Naomi R. Driesen, Genevieve Yang, Youngsun T. Cho, John D. Murray, David C. Glahn, Xiao-Jing Wang, John H. Krystal

**Affiliations:** ^1^Department of Psychiatry, Yale University School of Medicine, New Haven, CT, USA; ^2^NIAAA Center for the Translational Neuroscience of Alcoholism, New Haven, CT, USA; ^3^Abraham Ribicoff Research Facilities, Connecticut Mental Health Center, New Haven, CT, USA; ^4^Interdepartmental Neuroscience Program, Yale University, New Haven, CT, USA; ^5^Olin Neuropsychiatry Research Center, Institute of Living, Hartford Hospital, Hartford, CT, USA; ^6^Department of Psychology, Yale University, New Haven, CT, USA; ^7^Department of Psychology, Washington University in St. Louis, St. Louis, MO, USA; ^8^Department of Psychology, University of Ljubljana, Ljubljana, Slovenia; ^9^Department of Psychiatry, University of Zagreb School of Medicine, Zagreb, Croatia; ^10^Center for Neural Science, New York University, New York, NY, USA; ^11^Department of Neurobiology, Yale University School of Medicine, New Haven, CT, USA

**Keywords:** schizophrenia, pharmacology, functional connectivity, computational modeling, thalamus, NMDA receptors, glutamate

## Abstract

Neuropsychiatric diseases such as schizophrenia and bipolar illness alter the structure and function of distributed neural networks. Functional neuroimaging tools have evolved sufficiently to reliably detect system-level disturbances in neural networks. This review focuses on recent findings in schizophrenia and bipolar illness using resting-state neuroimaging, an advantageous approach for biomarker development given its ease of data collection and lack of task-based confounds. These benefits notwithstanding, neuroimaging does not yet allow the evaluation of individual neurons within local circuits, where pharmacological treatments ultimately exert their effects. This limitation constitutes an important obstacle in translating findings from animal research to humans and from healthy humans to patient populations. Integrating new neuroscientific tools may help to bridge some of these gaps. We specifically discuss two complementary approaches. The first is pharmacological manipulations in healthy volunteers, which transiently mimic some cardinal features of psychiatric conditions. We specifically focus on recent neuroimaging studies using the NMDA receptor antagonist, ketamine, to probe glutamate synaptic dysfunction associated with schizophrenia. Second, we discuss the combination of human pharmacological imaging with biophysically informed computational models developed to guide the interpretation of functional imaging studies and to inform the development of pathophysiologic hypotheses. To illustrate this approach, we review clinical investigations in addition to recent findings of how computational modeling has guided inferences drawn from our studies involving ketamine administration to healthy subjects. Thus, this review asserts that linking experimental studies in humans with computational models will advance to effort to bridge cellular, systems, and clinical neuroscience approaches to psychiatric disorders.

## Introduction

The human brain is a complex, dynamic system with computations occurring at several levels of organization, from individual synapses to networks that span multiple brain regions. These large-scale neural systems ultimately produce complex behaviors that are profoundly altered in the context of psychotropic drug administration or neuropsychiatric disease.

One example is schizophrenia – a common, multi-faceted, and heterogeneous neuropsychiatric syndrome (1) associated with disturbances in perception (2), belief (3), emotion (4), and cognition (5). A number of theoretical models of schizophrenia suggest that the clinical features of this disorder emerge from disturbances in neural connectivity and deficits in synaptic plasticity (6). Progress in explicating the pathophysiology of schizophrenia has been slowed by our limited understanding of the neurobiology of schizophrenia, shortcomings of animal models for this disorder, and the challenge of translating basic and clinical research approaches to this disorder. This knowledge gap has constrained our ability to develop new and more effective pharmacotherapies for schizophrenia (7), accounting for little change in the public health impact of this disease over the past two decades (8).

Disturbances in the structural and functional connectivity of the cerebral cortex are thought to be central to the neurobiology of schizophrenia (6) and are thought to impair the function of large-scale neural systems (9–13). Efforts have been made to reconcile these system-level observations with the cellular neuropathology of schizophrenia. One leading mechanistic model proposes that glutamate synaptic abnormalities associated with schizophrenia, mimicked in part by the effects of drugs that block the *N*-methyl-d-aspartate glutamate receptor (NMDAR) (7), disturb the local balance of excitation and inhibition and thereby contribute to alterations in large-scale neural system functional connectivity (14). This influential hypothesis is based on a key observation: sub-anesthetic doses of non-competitive NMDAR antagonists such as ketamine produce symptoms resembling those of schizophrenia in healthy humans (15). There is also growing evidence from pre-clinical (16), post-mortem (17), neuroimaging (18), and pharmacological experiments (15) illustrating that abnormal NMDA receptor function may be one pathophysiological mechanism occurring in people with schizophrenia. Alterations in synaptic function of gamma-aminobutyric acid (GABA) (19, 20) and dopamine (21) have also been implicated in schizophrenia, which likely affect local circuit computations (22). Collectively, this work provides insight into how disturbances in synaptic glutamate signaling (among other neurotransmitters) could contribute to producing schizophrenia symptoms (7). Nevertheless, there remains an important explanatory gap between mechanistic cellular-level hypotheses of schizophrenia and non-invasive neuroimaging studies that characterize the function of neural systems.

Functional neuroimaging has consistently revealed both region-specific and network alterations in schizophrenia across a number of cognitive measures (23). For instance, there is now substantial evidence suggesting profound alterations in networks supporting complex cognition in schizophrenia [e.g., working memory (WM) (24–26)]. This work has been complemented by a parallel focus on characterizing functional connectivity alterations in schizophrenia (27). Resting-state functional connectivity is based on the analysis of low-frequency fluctuations present in the blood-oxygenation-level-dependent (BOLD) signal (28, 29). These low-frequency fluctuations have been shown to be temporally correlated within spatially distinct but functionally related networks (30), establishing an intrinsic functional architecture (31) across species (32). The functional networks identified at rest also are correlated with other measures of structural and functional connectivity in healthy populations (33) and allow for characterization of distributed circuit abnormalities in neuropsychiatric illness (34, 35). Such approaches have been successfully applied to the study of schizophrenia and have increasingly revealed neuroimaging markers of this complex illness (12, 27, 36–38), which may be consistent with established theoretical models of this disease (39).

This review first focuses on resting-state functional connectivity MRI (rs-fcMRI) studies of schizophrenia that are providing distinct insights into cortical dysfunction associated with this disorder (37, 38). We highlight emerging connectivity strategies that deal with the complexity and heterogeneity of schizophrenia anatomy, physiology, and behavior (40). For instance, we articulate how data-driven tools that capture distributed connectivity abnormalities – such as global brain connectivity (GBC) – have the potential to avoid biases and identify network disturbances that traditional seed-based approaches may fail to detect. We next detail recent specific efforts to assay thalamo-cortical dysfunction in schizophrenia (38), long thought to be important to the clinical features of this disorder (41–43). We also discuss the potential utility of such data-driven approaches to discover variations in large-scale systems that may be altered across diagnostic categories (e.g., bipolar illness with psychosis and schizophrenia) and that may inform symptom-based or circuit-based diagnostic systems, such as the NIMH Research Domain Criteria (RDoC) (44–46).

The encouraging progress in understanding functional connectivity alterations in schizophrenia creates new opportunity to reconcile system-level findings with hypotheses emerging from molecular and cellular studies of this disorder. Failure to integrate these multiple levels of research undermines our understanding of the pathophysiology of this disorder and it will impede medications development. To this end, we next turn to studies using pharmacological models (47), such as the NMDAR antagonist ketamine, to test hypotheses related to the causes of circuit dysfunction in schizophrenia (18). Here we review a focused set of pharmacological neuroimaging (ph-fMRI) studies that utilized NMDAR antagonists to alter behavior and connectivity in healthy volunteers as a way to better understand schizophrenia (18, 48–54). These ph-fMRI investigations provide insight into how specific manipulations of synaptic function have effects that scale to produce both behavioral and system-level alterations that may be observed in patients. We articulate a set of directions for future studies that can capitalize on ph-fMRI as a tool to provide insight into specific illness-related mechanisms, especially when combined with advanced functional connectivity approaches.

**Figure 1:**
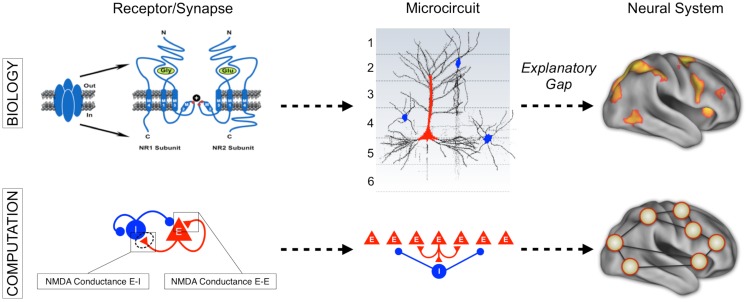
**Understanding complex mental illness from synapses to circuits to neural systems**. A major challenge facing the field of clinical neuroscience is building the links across levels of inquiry, from the level of receptors and cells, to microcircuits, and ultimately scale to the level of neural systems and behavior. At present, there is a vast explanatory gap across these levels in our understanding of psychiatric symptoms (top panel). Bridging this gap represents a major effort in explaining how alterations of specific mechanisms across neural systems may produce complex behavioral alterations seen in serious mental illness. This challenge is also exemplified by the National Institute of Mental Health Research Domain Criteria (RDoC) initiative (45) in order to map the biology across levels of inquiry onto behavior in a more systematic and data-driven way. We argue that computational modeling approaches (55) combined with additional experimental tools such as functional neuroimaging and pharmacology (47) offer one possible path toward this objective (bottom panel). We detail emerging efforts in functional connectivity work that may present a unique opportunity in this regard (59). Note: top left receptor figure was adapted with permission from Kotermanski and Johnson (60).

Finally, we briefly articulate the utility of neuroscience theory and computational models for iteratively guiding our pharmacological and clinical experiments (55, 56). We focus on one type of computational modeling that may hold promise in this regard – namely, biophysically realistic computational models that contain cellular-level detail necessary to characterize specific synaptic disturbances that may occur in disease states (56, 57). This level of biophysical detail can provide a vital opportunity to test both hypothesized synaptic alterations in schizophrenia (55) and also possible pharmacotherapies that may attenuate such disturbances (58). Despite possible advantages, we note some key limitations of these modeling approaches, pertaining to the constrained behavioral repertoire and neural architectures that are currently effectively modeled in this way, largely owing to gaps in our basic understanding of neurobiology that can constrain such models. Therefore, we articulate a key objective for the future of schizophrenia research: biophysically realistic computational models need to be systematically developed and scaled to the level of neural systems (59), to inform fMRI-level observations in schizophrenia as well as those observed following pharmacological manipulations in healthy humans (Figure 1). We argue that this approach could be especially productive for the study of functional connectivity in psychiatric conditions, with the ultimate aim of developing mechanistically derived biomarker predictions.

Building on these insights, we emphasize the need for efforts to translate our basic discoveries in neuroscience and cellular-level hypotheses in schizophrenia with system-level observations that may directly relate to the complex behavioral abnormalities observed in this illness. We highlight multiple complementary neuroscientific approaches, including recent clinical studies (37), pharmacological neuroimaging experiments (54), and theoretical/computational neuroscience approaches (18, 58) that can be synergistically harnessed to inform our understanding of underlying mechanisms and guide development of better pharmacotherapies for schizophrenia.

## Developments in Functional Connectivity Applications to Neuropsychiatric Disease

Nearly two decades ago Biswal and colleagues (61) demonstrated that coherent fluctuations in the BOLD signal exist across time and space, demarcating a functional network architecture in the human brain. Such early studies highlighted this phenomenon by focusing on the fluctuations between the left and right motor cortex. This key property of the BOLD signal has in turn generated a paradigm shift in non-invasive human neuroimaging and allowed for characterization of distributed neural networks across species in the absence of task-mediated effects (28–30, 32, 62). Importantly, a close correspondence between resting-state networks and task-based networks has been established within individual subjects and using meta-analytic techniques (31). The value of this approach has been enhanced by the subsequent emergence of analytic techniques that have provided insights into the component structure and regulation of distributed cortical networks (62). For instance, recent advances in graph-theoretical approaches have shown that cortical and subcortical networks can be segregated into unique community structures, providing a comprehensive data-driven mapping of human functional networks (63). Another set of studies has demonstrated the temporal non-stationary properties of the large-scale functional networks, delineating temporal functional modes of the human brain (64). In other words, there seem to be distinct and independent patterns of connectivity over both space and time. Although a comprehensive review of connectivity method developments is beyond the scope of this manuscript, we refer the reader to recent detailed reports on this topic (62). Here we specifically focus on select clinical applications in schizophrenia and bipolar illness.

Resting-state functional connectivity MRI approaches have been increasingly applied to neuropsychiatric illness (65). Use of this technique is built upon the hypothesis that specific neuropsychiatric conditions are brain disorders that affect computations across large-scale networks of regions or specific circuits in such a way that these alterations can be identified with functional connectivity measures. This hypothesis suggests that such disturbances in neural network function may reflect alterations in more basic cellular-level mechanisms. Thus, disturbances in molecular signaling or synaptic function would be hypothesized to scale and produce disturbances in large-scale neural systems (66). Such network-level disturbances in a given functional system may then reflect specific psychiatric symptoms (12, 27). This framework has guided the application of functional connectivity with the objective of identifying putative biomarkers via BOLD fMRI that could reveal neural system-level disturbances in neuropsychiatric conditions even in the absence of specific tasks. Such an approach may hold promise if its sensitivity and specificity is ultimately refined to the point of diagnostic utility.

Specific advantages of rs-fcMRI include: (i) speed, as the functional network architecture can be reliably assayed within 10 min of data acquisition (67), and perhaps even faster with recent advances in multi-band imaging technology (68, 69); (ii) possible cost-effectiveness. Task-based studies may require longer time for data acquisition, depending on the precise paradigm being imaged. (iii) Lack of performance confounds, which likely affects nearly every cognitive activation task when applied to a psychiatric population (70). (iv) In addition, when coupled with techniques such as GBC or independent component analyses (ICA), rs-fcMRI studies allow the unbiased, simultaneous examination of all neural networks (see below for more discussion). All of these features suggest that rs-fcMRI may be a feasible method for eventually guiding and tracking symptoms, illness progression and possibly response to pharmacotherapies and/or behavioral treatments.

The approach itself, however, is not without limitations. For instance, the size of the correlation coefficient is often used to examine the strength of coupling between different network nodes (71). While this is an analysis-level consideration, the majority of published studies use this technique. This approach may not be ideal for examining shared versus non-shared variance between regions and may obscure more complex differences that could occur in psychiatric conditions (71). For instance, it may be important to uniquely identify functional connectivity alterations that truly reflect altered neuronal communication between two network nodes, as opposed to the influence of a third region on both (a correlation-based approach could not disambiguate these possibilities). It is also important to emphasize that functional connectivity, as measured at rest, is purely correlational and therefore can only be used to make tentative causal inferences. That is, if there is evidence for an alteration in a given circuit, this alteration could be predictive of disease onset or could be a consequence of some upstream physiological alteration. Moreover, there are additional complications involved in the study of neuropsychiatric illness concerning what is a primary deficit, as opposed to a compensation or treatment effects. These potential confounds underscore the complementary value of causal experimental designs involving pharmacologic, neuro-stimulation, or cognitive manipulations of circuit activity. Finally, there are still notable differences in the methods used to analyze rs-fcMRI data. For instance, one ongoing controversial issue relates to removal of the global signal (i.e., global signal regression, GSR) from functional connectivity data (72, 73). This step effectively regresses out variance associated with global brain fluctuations, which can be large in magnitude and can perhaps obscure certain real functional relationships (74). Nonetheless, this step effectively shifts the mean of the connectivity distribution, resulting in a portion of correlations being moved into the negative range – thus spuriously inducing at least some anti-correlations. There is convincing evidence from electrophysiology experiments using animals that anti-correlations indeed exist (75, 76). However, GSR could still complicate some aspects of between-group comparisons and the interpretations of clinical studies (73), especially when examining system-level relationships across groups. Therefore, future studies should carefully continue to consider the possible impact of this step on between-group differences in clinical rs-fcMRI studies.

Another methodological issue pertains to how networks are identified. Many early functional connectivity studies both in healthy adults and in clinical populations used a seed-based approach, whereby the correlation from a given region of interest is examined with all other voxels in the brain. This approach has also been complemented by more complex graph theoretical and network-based methods (77). Seed-based approaches inherently assume a consistent difference across a set of regions in a clinical condition. However, the pattern of dysconnectivity for a single region could indeed be more variable both across subjects and within regions. Such spatial variability in functional networks across subjects may present a limitation of seed-based approaches when applied in studies of clinical populations (11, 40). This may be the case in complex mental illnesses such as schizophrenia. Therefore, detecting more complex patterns of dysconnectivity requires new approaches taking into account these confounds (78–80).

**Figure 2:**
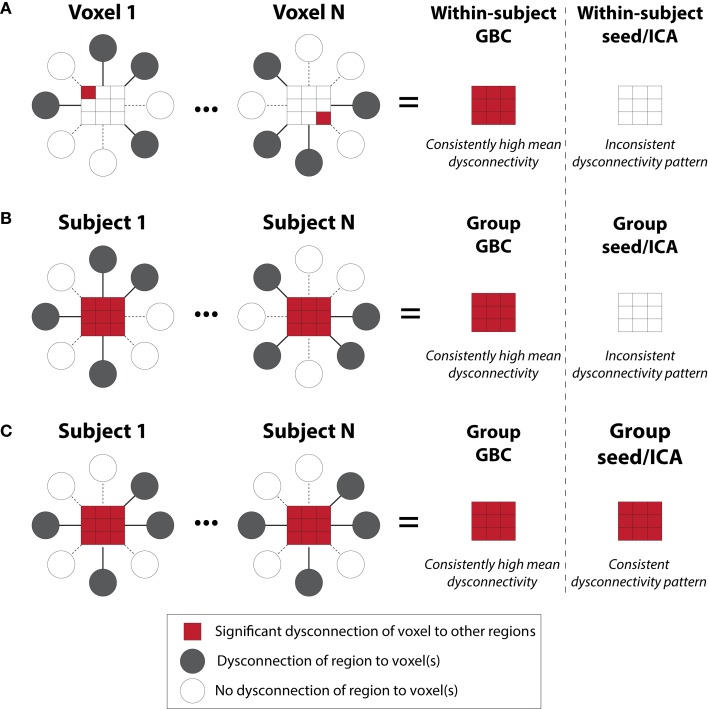
**Global brain connectivity identifies globally dysconnected regions despite substantial within or between subject variability**. We hypothesized that some brain regions may have global variable disruptions in functional connectivity, which may contribute to individual differences in symptom severity in brain disorders. GBC can be used as a data-driven approach to search for such regions. GBC is computed as the average connectivity of each voxel to all others. Gray circles indicate regions with altered connectivity to the depicted red voxels. **(A)** GBC will identify a region with consistent global dysconnectivity even when there are substantial differences in connectivity patterns within the region. Seed or ICA approaches would be unable to identify dysconnectivity in such a region because of inconsistent dysconnectivity patterns across neighboring voxels. **(B)** GBC, unlike seed or ICA approaches, will identify a region with consistent global dysconnectivity even when there are substantial individual differences in connectivity across subjects. This allows the identification of regions with many individual differences in connectivity, which might correlate with individual differences in symptoms. **(C)** GBC is also sensitive to consistent group differences in connectivity that might be identified using seed or ICA approaches, though GBC might be more sensitive due to pooling of results for both consistent and inconsistent dysconnectivity. GBC, global brain connectivity; ICA, independent component analysis. Note: figure adapted with permission from Cole and colleagues (11).

New methods are needed to detect more complex patterns of disorder-related disturbances in connectivity (80), illustrated by the GBC approach (11, 35, 40, 81). The GBC technique is specifically designed to consider connectivity from a given voxel or (area) to all other voxels (or areas) simultaneously by computing either average connectivity strength (weighted GBC) or by counting the number of connections above a given connection strength (un-weighted GBC). Thus, this approach is data-driven and unbiased as to the location of connectivity disruption. That is, unlike typical seed-based approaches, GBC requires only the seed region to be relatively consistently located across subjects, while the target regions can vary substantially across subjects (Figure 2). For example, if a given area is perturbed in its functional connectivity consistently, irrespective of the overall network spatial configuration where the perturbation is, GBC will remain sensitive to this alteration. Further, unlike typical seed-based approaches, GBC involves one statistical test per voxel (or ROI) rather than one test per voxel-to-voxel pairing – substantially reducing multiple comparisons (e.g., 30,000 rather than ~900 million tests). These two improvements over typical seed-based approaches can dramatically increase the chance of identifying group differences in connectivity, or individual differences in connectivity correlated with behavioral symptoms (11, 35, 40, 81). Indeed, consistent with these proposed advantages, GBC has now been applied to identifying regions with large-scale disruptions in functional connectivity across a variety of mental illnesses, such as schizophrenia (11), bipolar disorder (35), and obsessive-compulsive disorder (OCD) (82). GBC is explicitly designed to address questions about brain connectivity that are qualitatively distinct from traditional seed-based functional connectivity analyses. For instance, areas of high GBC are “hubs” of connectivity in the brain that are maximally functionally connected with other areas and may play a role in coordinating large-scale patterns of brain activity (40). In that sense, a group difference in GBC may reflect areas and networks in which the large-scale coordination of information processing is affected in the disease state. For instance, decreased GBC may reflect decreased participation of a brain region in broader networks. Conversely, increased GBC may suggest a pathological broadening or synchronization of functional networks. Related to this point, we have now published a manuscript examining distributed networks in OCD using GBC where specific striatal and orbitofrontal circuits have been implicated. Yet, GBC remains sensitive to alterations in neuropsychiatric conditions that may actually be associated with more focal deficits (which may be the case in OCD versus schizophrenia). In that sense, GBC is not necessarily more useful for “global” versus “restricted” deficits, but rather dysconnectivity to a given “hub” region that may be affected in its participation in wide-spread neural networks. These properties of GBC – detection of regions with large-scale functional connectivity disruptions along with tolerance for individual differences – makes this method particularly powerful for identifying regions with consistently large and distributed disruptions that have functional consequences reflected in individual differences in psychiatric symptoms. Continued refinement of graph-theoretical data-driven metrics such as GBC may allow a powerful path forward for delineating biomarkers within and across diagnostic categories.

Another data-driven method that has increasing applications in psychiatric neuroimaging involves ICA of BOLD signal fluctuations at rest. Here ICA provides a tool to identify spatially (78, 83) or temporally (64) independent modes of brain function. ICA has been successfully used to identify distributed network abnormalities in both schizophrenia and bipolar illness (84). In particular, temporal ICA (64) may provide a novel and powerful method to assay the non-stationary properties of distributed neural systems across different clinical conditions. It remains to be systematically tested if recently defined temporal functional modes exhibit alterations in neuropsychiatric illness. One possibility, given wide-spread neurotransmitter disruptions in schizophrenia, is that the temporal functional modes are substantially more non-stationary in this illness. Powerful new acquisition sequences [e.g., multi-band imaging (68)], that allow much finer temporal sampling, will provide critical technological innovations at the level of neuroimaging acquisition with direct clinical applications, in particular when using ICA-type measures.

Collectively, the continued success of these methods suggests that ongoing refinement of tools to examine functional connectivity remains vital for development of biomarkers for psychiatric illness. Next, we turn to specific findings in the field of schizophrenia and bipolar research. We detail recent methodological advances in connectivity work that have been directly applied to better understand network disruption in these psychiatric conditions.

## Emerging Evidence Suggests Large-Scale Dysconnectivity in Schizophrenia

There are now a number of influential hypotheses suggesting that, at its core, schizophrenia may be a disorder of large-scale neural connectivity (6, 85), i.e., “dysconnectivity.” Here we refer to dysconnectivity as an alteration in neural communication that specifically produces behavioral pathology, as opposed to a simple alteration in functional connectivity that deviates from the norm. The models proposing such dysconnectivity range from theoretical to formal computational hypotheses (42, 43, 66, 86–88). This work has inspired the search for connectivity biomarkers for schizophrenia and it broadly includes: (i) studies that use rs-fcMRI, that is investigations that study the intrinsic properties of BOLD signal fluctuations to delineate system-level alterations in psychiatric illness (27); (ii) studies that examine task-based dysconnectivity, that is alterations in connectivity during specific task contexts; (iii) pharmacological studies of individuals at rest or performing tasks, which we discuss in the upcoming section. While the literature on task-based connectivity has offered important clues regarding network alterations during specific cognitive processes (e.g., WM) (27, 89, 90), here we specifically detail emerging efforts to use rs-fcMRI to define biomarkers for schizophrenia. This is not to say that characterizing functional connectivity during cognitive processing is not important. In fact, such studies represent a vital effort to move our understanding beyond activation-based hypotheses regarding cognitive deficits in schizophrenia or regional hypotheses of specific symptoms. In the future, task-based functional connectivity studies will ultimately aid our understanding of network dysfunction with respect to specific symptoms or specific cognitive processes known to be profoundly affected in schizophrenia (e.g., WM) (26). Additionally, task-based studies of functional connectivity provide an important assurance that observed differences in resting-state functional connectivity do not merely reflect differences in the dynamics and content of mental processes subjects might engage in during rest, but rather stable disruptions in functional connectivity that persist across mental states (90). However, rs-fcMRI can be used to identify neural-system alterations even more broadly. With that in mind, we argue that rs-fcMRI offers a unique tool to identify distributed neural system alterations, extending beyond a given task context. Moreover, as noted above, this approach in particular bypasses task performance confounds which plague the task-based activation literature (70) and such concerns will likely extend to task-based connectivity work.

Despite its promise, a major obstacle in delineating successful neural markers for schizophrenia (or any other severe neuropsychiatric disease) using connectivity (or any other approach for that matter) has been the complexity of this illness and the vast range of behaviors it affects (91), as well as the complex temporal dynamics of its progression. That is, the apparent clinical complexity of schizophrenia is a major obstacle to biomarker development that may apply to all patients. In addition, the associated clinical heterogeneity (i.e., differences in symptoms across patients), the presence of numerous comorbidities and environmental modifiers, the ubiquitous confound of long-term antipsychotic treatment, and the wide range of affected behaviors collectively reduce the likelihood that a single biomarker would be applicable to the entire syndrome. Moreover, many researchers and clinicians would argue that schizophrenia is a theoretical construct used to label co-occurring symptoms whereby it may be challenging (or perhaps impossible) to map out a reliable and replicable marker of the entire syndrome in a parsimonious way [although theoretical models of the disease often consider it as one entity (66)]. We argue that functional connectivity tools offer a useful path forward in this regard by providing methods to test specific large-scale dysconnectivity patterns in relation to this heterogeneity that may be otherwise difficult to capture.

Certain studies using rs-fcMRI have, however, attempted to deal with clinical heterogeneity by focusing on the relationship between altered functional connectivity in specific pathways and linking those pathways to particular features of schizophrenia. For instance, studies by Hoffmann and colleagues have focused on better understanding alterations in connectivity specifically associated with auditory hallucinations (92), which many patients experience (1). They found compelling evidence for disruptions between regions associated with auditory processing. Specifically, they found that when they seeded Wernicke’s area, there was significantly greater functional connectivity to Brodmann’s areas 45/46 among hallucinating patients compared with non-hallucinating patients. In a subsequent analysis, they reported that the functional connectivity within a functional loop including the Wernicke’s area, inferior frontal gyrus, and putamen was robustly greater for hallucinating patients compared with non-hallucinating patients. Vercammen and colleagues also found that patients with schizophrenia evidenced attenuated functional connectivity between the left TPJ (temporo-parietal junction) and the right Broca’s area (93). These are examples where targeted seed-based approaches may identify alterations in circumscribed circuits associated with specific symptoms.

Another set of emerging studies have studied the “salience” and “control” systems, focused on striatal and insular dysconnectivity in schizophrenia (94, 95), particularly in relation to the “aberrant salience” hypothesis (96, 97). Briefly, the “aberrant salience” hypothesis has been linked to abnormal striatal dopamine function, suggesting that during psychotic states patients have a higher likelihood of forming inappropriate associations and respond excessively to random neutral events. Related to this issue a study by Tu and colleagues examined whether schizophrenia is associated with functional connectivity alterations within the cingulo-opercular (CO) network specifically (98). They identified significantly reduced functional connectivity in the bilateral putamen for patients with schizophrenia, which was related to cognitive performance in patients. The authors concluded that schizophrenia is associated with disconnection within cortico-striatal circuits. A complementary study by Moran and colleagues (99) focused on the anterior insula as a key node involved in modulation of distributed neural systems (as part of the CO system). The authors tested for disruptions in the functional relationships between the insula and control/default networks in patients with schizophrenia. Similarly, they examined a relationship between these network disturbances and cognitive deficits. Consistent with *a priori* predictions, Moran and colleagues found strong support for the disrupted relationship between the anterior insula and the control-executive and default-mode networks in schizophrenia, which again was predictive of cognitive performance. Most recently, a study by Palaniyappan and colleagues (100) also focused on the relationship between the salience (insular) system and the executive [lateral prefrontal cortex (PFC)] networks in schizophrenia. They explicitly tested for evidence of disrupted directional influence across the networks. In other words, similar to Moran and colleagues, they used an auto-regressive technique (i.e., Granger causality) to examine both feed-forward and reciprocal connectivity between the aforementioned networks. The authors reported significant differences in patients with regard to time-lagged functional relationships between executive and insular systems. The authors conclude that this “breakdown” in directional functional relationships may reflect aberrant processing of novel salient information. These studies provide compelling emerging evidence that there may indeed exist causal breakdowns across functional networks in schizophrenia. Nevertheless, it is important to note that lag-based causality measures in the context of BOLD signal analyses have not been without controversy (101–103), primarily because of systematic difference in the hemodynamic response function lags across areas. Given these concerns, the use of Granger causality in rs-fcMRI studies needs to be interpreted with caution [see Ref. (104) for a more detailed treatment of using auto-regression techniques with the BOLD signal]. Future studies using concurrent electrophysiology/fMRI protocols could provide convergent evidence to address issues regarding temporal dependencies across cortical networks when analyzing the BOLD signal.

We discussed studies that focused on either specific regions or networks that may be abnormal in schizophrenia. An alternative tactic, precisely because of the complexity of this illness, is to use data-driven methods to study large-scale connectivity alterations that may be difficult to pinpoint *a priori*. A number of prominent and well-established models of schizophrenia neuropathology implicate profound disruptions in PFC function (105), likely stemming from a confluence of glutamate, GABA, and dopamine alterations that could jointly affect PFC function (7, 19, 106–108) (see Box 1). One area that has been repeatedly implicated in schizophrenia neuropathology is the dorso-lateral PFC (23). However, this evidence has largely been marshaled through task-based studies such as those examining WM deficits in this illness (70). A deficit in task-evoked computations of a given region does not necessarily guarantee that the same node will show other forms of functional “dysconnectivity.” Moreover, such evidence does not guarantee that this same area may be disrupted in its functional connectivity in the absence of a task (i.e., during resting-state). Nevertheless, PFC functional deficits have been considered a hallmark feature of the illness. Therefore, one promising approach is to examine global PFC dysconnectivity in a data-driven way.

Box 1 **Glutamate versus dopamine: upstream versus down-stream mechanisms in schizophrenia.**

It is increasingly acknowledged that schizophrenia is associated with disturbances in multiple neurotransmitter systems, including alterations at the cortical microcircuit level in glutamate and γ-aminobutyric acid (GABA), as well as disturbances in dopaminergic neurotransmission along striatal-thalamic-cortical pathways (7, 106, 109). Additional studies have also implicated the glial system as possibly compromised in schizophrenia (110, 111) and other neuropsychiatric conditions (112). However, it remains unknown whether dopamine or glutamate alterations are the upstream causes or down-stream consequences of the disease process (109). Addressing this question has important implications for appropriately constraining computational models, both at the microcircuit level (58) and expanding those mechanisms to the system-level (18). There are two broad possibilities to consider: (i) there may be dissociable groups of patients each associated with a primary abnormality in one of the broad neurotransmitter systems, but as a consequence there may be secondary abnormalities in the other system due to shared pathways and functional loops (43, 88). (ii) One alternative possibility is that there are always primary alterations in only one of the two systems, followed by alterations in the other. Disambiguating between these causal possibilities can have important implications for developing optimized targeted pharmacotherapies for specific patient groups, which is a likely possibility given the heterogeneity of the illness. By extension, resolving these issues may have implications for optimized pharmacotherapies for a given phase of illness [if initial stages of schizophrenia are primary associated with hyper-glutamatergic neurotransmission (113)]. Also, these two possibilities have important implications for the utility of pharmacological models of psychosis (e.g., amphetamine versus the NMDAR antagonist challenge studies in healthy volunteers).

Here it is important to consider some key differences between the dopamine and glutamate hypotheses in schizophrenia in relation to pharmacological findings and their therapeutic effects: (i) dopaminergic medication has not proved successful at ameliorating the full range of impairing symptoms in schizophrenia, particularly cognitive deficits (26); (ii) pharmacological models of schizophrenia targeting the dopamine system (e.g., amphetamine challenge) have typically produced a clinical profile marked by acute psychosis, as opposed to a broader range of symptoms produced by pharmacological agents targeting the glutamate system (15, 114, 115). Therefore, while it is certainly important to acknowledge dopamine as a key component of disrupted neurotransmission in schizophrenia (21, 116, 117), it remains to be determined if dopamine is indeed a down-stream cause or a consequence of primary disruptions in glutamate (118). Relatedly, irrespective of cause or consequence arguments it will vital to consider the diversity of DA receptors (D1 versus D2) and their respective sites of influence in cortex (22, 119, 120) versus striatum (108), which in turn generates important constraints for computational modeling studies that incorporate dopaminergic and glutamatergic signaling mechanisms.

**Figure 3:**
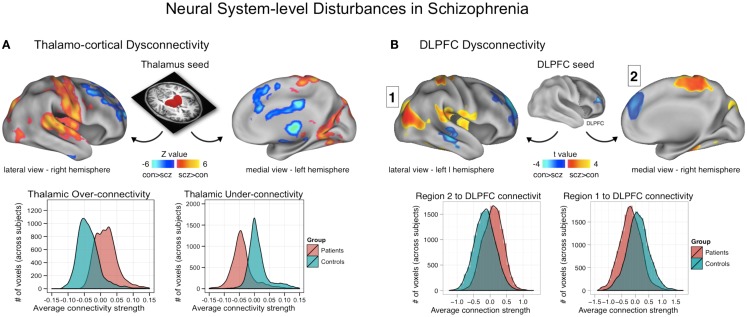
**Neural system-level dysconnectivity in schizophrenia – emerging biomarkers**. **(A)** Results from a recent large connectivity investigation examining thalamo-cortical connectivity alterations in 90 patients diagnosed with schizophrenia relative to 90 matched healthy controls (38). Anticevic and colleagues found robust alterations in thalamo-cortical information flow in schizophrenia, whereby sensory-motor cortical regions showed over-connectivity in schizophrenia (regions shown in yellow-red), but prefrontal-striatal-cerebellar regions showed under connectivity in schizophrenia relative to controls (regions shown in blue). Anticevic and colleagues fully replicated this pattern in an independent sample. Woodward and colleagues, using complementary approaches, found highly comparable effects (37). **(B)** A similar pattern of over/under connectivity was identified in patients with schizophrenia when using a DLPFC seed region identified via GBC; as with the thalamic seed, there was increased coupling with sensory (posterior regions, shown in yellow-red) but reduced connectivity with prefrontal and other higher-order temporal regions (shown in blue). This over/under pattern recapitulated qualitatively the observations found for the thalamic analysis in **(A)**, as shown in the distribution plots on the bottom of each panel. Note: figures adapted with permission from Anticevic and colleagues (38) and Cole and colleagues (11).

As articulated above, the GBC functional connectivity approach is specifically designed to test the hypothesis that a given functional brain region has altered coupling with the rest of the brain (or a large anatomical portion of the brain, such as the PFC). To test the efficacy of this approach, Cole and colleagues (11) used a restricted GBC (rGBC) approach focused on PFC in particular in a sample of patients with chronic schizophrenia relative to demographically matched healthy comparison subjects. Cole and colleagues reported that bilateral regions centered on the lateral PFC showed reductions in their PFC rGBC, suggesting that these regions have profound alterations in their connectivity patterns with the rest of PFC. To further test the relationship between identified PFC rGBC alterations and cognitive deficits, Cole and colleagues quantified the relationship between IQ and the PFC global connectivity alterations. The authors found that greater connectivity between the identified lateral PFC and the rest of PFC predicted better cognitive performance, suggesting that a lower index of global PFC coupling may in part relate to cognitive deficits in schizophrenia. Moreover, the authors additionally examined the pattern of whole-brain coupling of the discovered right lateral PFC region using seed-based techniques. The authors found that patients diagnosed with schizophrenia showed increased coupling with sensory cortices (Figure 3B, posterior regions, shown in yellow-red). Patients also showed reduced connectivity with prefrontal and other higher-order temporal regions (Figure 3B, shown in blue). Collectively, this initial report demonstrates that data-driven functional connectivity approaches could identify regions previously implicated in schizophrenia using task-based methods. Moreover, these identified areas showed robust and complex alterations of connectivity with the rest of the brain and importantly, related to observed symptoms and other cognitive measures.

Similar data-driven efforts have been applied to study global connectivity alterations in other psychiatric conditions. Specifically, Anticevic and colleagues extended the approach to study bipolar patients with and without a history of psychotic symptoms (35). While examining neural system-level disturbances in schizophrenia is a vital objective, psychosis occurs across a number of diagnostic categories. For instance, many bipolar patients experience frank psychosis (121). Given this clinical observation, some studies divide bipolar patients based on the presence or absence of psychotic symptoms (122, 123). In that sense, there may be somewhat limited utility in predefined diagnostic boundaries for understanding variation in neural circuits that could possibly be affected across neuropsychiatric conditions (45). Recognizing this limitation, there are emerging efforts to map common aspects of neural dysfunction across diagnostic categories.

Focusing on bipolar illness, Anticevic and colleagues examined the possibility that there are similar abnormalities in bipolar illness with a history of psychosis to those found in schizophrenia. Consistent with this hypothesis, Anticevic and colleagues found that bipolar illness is indeed associated with altered PFC rGBC specifically in a medial prefrontal/ventral cingulate region implicated in regulation of emotion (35). Importantly, this effect was primarily driven by a presence of psychosis history, and the magnitude of the observed PFC rGBC disruption was correlated with the severity of prior psychosis. This effect, however, was obtained in euthymic bipolar individuals (as opposed to symptomatic schizophrenia patients above), which suggests that at least some alterations in global connectivity may be a stable (trait) feature of the illness and may not necessarily only manifest in overtly symptomatic individuals. In that sense, data-driven functional connectivity may provide a tool to examine individual variability in both current and/or lifetime symptoms. Such data-driven approaches can also be used to establish alterations in functional neural architecture over time – namely, whether the observed disruptions in schizophrenia and bipolar illness change longitudinally as the illness progresses. This possibility is consistent with prominent neurodevelopmental models of severe psychiatric illness (14, 124–126), which suggest that there may be profound functional changes along the illness progression, to which these tools may be sensitive.

While promising, a key challenge facing data-driven approaches will be to establish whether identified effects consistently replicate across sites and samples. It may be possible that illness sample heterogeneity, illness stage, symptom severity, anatomical heterogeneity, or other factors will critically impact the patterns of data-driven effects across studies and samples. That is, it may be possible that different foci are detected as showing disruptions in different samples. Therefore, it will be critical to determine which of the identified data-driven global connectivity alterations are stable and replicable (perhaps capturing some core disturbances) and which alter as a function of other variables (perhaps capturing state effects). Such ongoing data-driven efforts should also continue to capitalize on recent advances in graph theory (80) as well as neuroimaging acquisition-level improvements (64, 69, 127) which could jointly improve the sensitivity of resting-state connectivity-derived metrics.

As described above, the PFC has been repeatedly implicated in schizophrenia neuropathology. However, despite data-driven efforts to map PFC dysfunction, the PFC remains a challenge in the study of schizophrenia, owing largely to the complex individual differences in function and anatomy of the PFC. Therefore, to ultimately establish a viable large-scale, brain-wide marker of neural alterations in schizophrenia, an alternative approach can be taken. One possible path is to start from the thalamus as a key region of interest (42). That is, while the PFC has classically been implicated in schizophrenia neuropathology, recent studies highlight disturbances in additional neural foci. In particular, the thalamus has emerged as an important disrupted locus in schizophrenia. While we presented some arguments against using seed-based approaches, the thalamus may present a unique opportunity in this case. Here we argue for the utility of specifying a seed when there is a specific theoretically guided hypothesis implicating a given area in the disease process. In this case, examining thalamic connectivity using rs-fcMRI in schizophrenia capitalizes on several key aspects of this subcortical region: (i) the thalamus is topographically connected to the entire cortex (128, 129), and may therefore represent a node particularly sensitive to network-level disturbances (42); and (ii) the thalamus contains anatomically and functionally segregated nuclei readily identifiable via neuroimaging (130) thus providing a lens for examining parallel yet distributed large-scale connectivity disturbances in neuropsychiatric disease.

Consistent with this view, a large body of evidence implicates significant thalamo-cortical communication disturbances in schizophrenia neuropathology (41, 42, 131–134). In fact, a fundamental aspect of large-scale brain organization preserved across mammalian species is thalamo-cortico-striatal sub-circuits that are thought to integrate various functions such as emotion processing and motor output (135–138). Such circuits may become profoundly dysregulated in schizophrenia, and the thalamus, as an organized hub of cortical and subcortical connections, may be especially sensitive to such dysregulation. Essentially, the thalamus serves as a nexus for parallel circuits through which diverse cortical and subcortical functions are integrated and distributed throughout the cortical mantle (128, 139, 140). These thalamic circuits have been implicated in schizophrenia pathophysiology on the basis of neuropathology studies (88, 141–143), pre-clinical lesion models (144, 145), structural imaging studies (146, 147), and computational models (43). Moreover, thalamic abnormalities are repeatedly implicated in sensory gating (148, 149) and filtering disruptions (150, 151) associated with this disorder (42). Indeed, one prominent model of schizophrenia neuropathology is centered on the thalamus as a key hub of disrupted computations in this illness. This “cognitive dysmetria” hypothesis articulates a distributed disruption in information processing across widespread cortical and subcortical nodes (39). In their seminal theoretical piece, Andreasen and colleagues argue that in order to explain the full range of schizophrenia symptoms, the field has to move away from region-specific models, but rather consider a distributed processing deficit, which could also parsimoniously explain a computational abnormality in a given node of a distributed complex system. In that sense, the thalamus is a uniquely positioned set of nuclei that communicates with virtually every cortical territory and is likely to be profoundly affected in schizophrenia.

Building on theoretical, pre-clinical, and anatomical work, recent studies of functional connectivity have begun to map thalamo-cortical alterations in schizophrenia. The first study to do so, by Welsh and colleagues (152), focused on the medio-dorsal nucleus of the thalamus as a seed region. This focused approach is justified given that specific thalamic nuclei in schizophrenia may show particularly profound functional connectivity disruptions. The medio-dorsal nucleus projects heavily to PFC regions (153, 154), and is thought to be compromised in schizophrenia (142, 144, 155). Welsh and colleagues found lower connectivity between the medio-dorsal nucleus and the PFC in patients with schizophrenia relative to healthy comparison subjects. However, this early investigation was based on a very small sample and could therefore be limited in its ability to provide conclusions regarding more subtle disruptions elsewhere. Moreover, Welsh and colleagues could not provide information regarding additional thalamic nuclei given the explicit focus on the medio-dorsal nucleus. In a subsequent study, Woodward and colleagues employed a substantially more powered sample (*N* = 62) and extended the approach to other thalamic nuclei (37). The authors used a parcellation scheme of thalamo-cortical connections at rest provided by Zhang and colleagues (129). In the study by Zhang and colleagues, cortical areas were clustered into subdivisions that exhibited unique functional connectivity with distinct thalamic nuclei based on the similarities in resting-state BOLD signal. Woodward and colleagues harnessed this segmentation scheme to test the hypothesis that unique thalamo-cortical circuits may show different patterns of disturbances in schizophrenia. Strikingly, Woodward and colleagues found that compared to healthy controls, the thalamic segmentations associated with the PFC showed reduced connectivity in schizophrenia. In contrast, cortical territories centered on sensory-motor regions showed increases in thalamic coupling in schizophrenia. This evidence suggests that there exists a profound alteration in thalamo-cortical information flow in schizophrenia but one that seems to follow an anatomical dissociation between sensory and higher order association regions. Building on this robust evidence for thalamo-cortical alterations in schizophrenia it is important to provide information about the specific connections being affected. Specifically, the cortical parcellation scheme, while a powerful initial demonstration, did not allow for examination of the cerebellum for instance. Cerebellum is a structure that, like the striatum, has projections to the cortex by way of the thalamus (156), and has been implicated in schizophrenia pathophysiology (39). Lastly, perhaps due to restricted power, the authors could not examine subtle relationships between symptoms and identified dysconnectivity.

A subsequent report by Anticevic and colleagues examined thalamo-cortical dysconnectivity in 90 patients diagnosed with schizophrenia relative to 90 matched healthy comparison subjects (38). The key objective of this investigation was to determine if thalamo-cortical disturbances span across diagnostic boundaries that share similar symptoms. This cross-diagnostic extension directly informs the objectives articulated by the NIMH RDoC initiative, which aims to develop biomarker-driven diagnostic systems (45). First, the authors replicated the core findings by Woodward and colleagues, demonstrating that schizophrenia is associated with increased coupling between the thalamus and all sensory-motor cortices. In contrast, frontal-striatal-cerebellar nodes showed reduced coupling with the thalamus in schizophrenia relative to healthy comparison subjects (Figure 3A). Both patterns were fully replicated in an independent and smaller sample of patients. Critically, further analyses demonstrated that these two sources of disturbance were functionally related. That is, those patients with the highest sensory-motor-thalamic coupling also showed the lowest prefrontal-striatal-cerebellar-thalamic coupling. This effect was most prominent for thalamic clusters centered on the medio-dorsal nucleus with known dense projections to the PFC, ruling out the possibility of pan-thalamic dysconnectivity that is uniform. Furthermore, the magnitude of the sensory-motor-thalamic over-connectivity was correlated with PANSS symptom severity across patients, confirming its functional relevance. The magnitude of this correlation, however, was small (*r* = 0.23) – indicating that the observed pattern explains only a small portion of the variance in symptom variation across subjects. An alternative possibility, given that the majority of patients were quite symptomatic, is that the small magnitude reflects a restricted range in symptoms whereby there was little variability in symptom severity across the patient sample.

The identified thalamic dysconnectivity was successfully used for diagnostic classification via multivariate pattern analysis (MVPA) (157) with high levels of sensitivity and specificity across both the discovery and replication samples. This implies that the identified dysconnectivity patterns, while not yet qualifying for a robust biomarker, may be refined and used to predict risk and assess treatment response. In particular, there are major ongoing improvements in neuroimaging acquisition (127) and processing (69) technology as a direct consequence of the Human Connectome Project (158) that can enable future studies to iteratively refine neuroimaging approaches. Such studies can focus on the identified patterns to ultimately improve methods and fine-tune the identified patterns for biomarker use.

Lastly, the authors found that the bipolar illness sample exhibited an “intermediate” pattern of disturbance such that the patterns of thalamo-cortical connectivity were “shifted” relative to healthy comparison subjects but not as severely altered as those identified in schizophrenia. This finding in particular offers promise for using neuroimaging markers to inform our understanding of shared disturbances in the underlying biology that cut across diagnostic categories (123). The next step will be to understand how such shared neural system-level “endophenotypes” (159, 160) map onto co-occurring behavioral disturbances (e.g., psychosis) as well as onto possibly shared alterations at the level of cortical microcircuits (which we discuss in the last section).

Most recently, Klingner and colleagues provided convergent results from a sample of 22 patients diagnosed with schizophrenia and 22 matched comparison subjects (161). In their study they separately examined the left and right thalamic seeds. They found robust evidence for increased thalamic connectivity with bilateral sensory-motor and auditory cortices. The authors conclude that their results suggest a possible “lack of thalamic control on motor/sensory information processing resulting in increased (and less filtered) forwarding of information to the prefrontal cortex.” This hypothesis is consistent with findings from the two earlier and larger studies (37, 38). Interestingly, Klingner and colleagues did not observe notable reductions in thalamic coupling with frontal-striatal-cerebellar nodes in schizophrenia, reported by both aforementioned groups. There are at least two possible interpretations for this difference: (i) the sample size in the Klingner study was much smaller and possibly underpowered to find both sets of patterns (although the effect size analysis and replication analyses by Anticevic and colleagues argues against this possibility); and (ii) Klingner and colleagues may have employed techniques, such as using GSR as a preprocessing step, that could have led to different results (73). It remains to be systematically determined what the true contribution of the global signal is in these analyses, especially in the patient groups. One possibility is that the global signal carries biologically meaningful information regarding cortical-thalamic disruptions in schizophrenia that needs to be carefully characterized. Methodological issues notwithstanding, this emerging body of work strongly and consistently implicates disruptions in thalamo-cortical information flow as a neural system marker in schizophrenia.

While this initial progress in mapping thalamo-cortical disturbances in schizophrenia represents a promising advance in psychiatric neuroimaging research, there are still fundamental gaps in our understanding of how such findings relate to neuropathological mechanisms of this illness. There are a number of future directions that the field should pursue to better understand these observations. For instance, it remains unknown why there are dissociable disturbances across thalamic nuclei in schizophrenia. Future studies focused more exclusively on the medio-dorsal nucleus versus, say, the pulvinar (known to be more involved in visual processing) could begin to explain mechanism behind these differences. Although the original study by Welsh and colleagues provides clues here, follow-up studies with more power that focus on the medio-dorsal nucleus could provide finer-grained information regarding its patterns of dysconnectivity with the PFC.

Another complex issue that is not adequately resolved by any of the noted investigations relates to medication effects. For instance, there are considerations of medication dose, type of medications (given that patients are often treated with multiple drugs from different medication classes), and possible systematic differences in the medications received by patients carrying the diagnoses of schizophrenia, schizoaffective, and bipolar (e.g., mood stabilizers and anti-depressants). While all studies address this issue statistically to a certain extent (by computing chlorpromazine equivalents and then co-varying for the medication dose), future studies in un-medicated patients are needed. It is possible, however, that medication may not necessarily be a confound in this case – instead, antipsychotic medication could actually stabilize thalamo-cortical dysconnectivity. Studies explicitly aimed at testing medication effects on connectivity could provide more detailed insight into this issue. It also remains unknown if the identified patterns of large-scale thalamo-cortical dysconnectivity are characteristic only of chronic stages of schizophrenia or whether they already appear in the prodromal or early stages of the illness. Establishing the link between identified thalamo-cortical dysconnectivity and illness progression remains a vital effort to inform the viability of this marker for predicting and/or tracking risk and progression of the disease. While one of the studies noted above provides a functional link between sensory-motor-thalamic over-connectivity and PANSS symptoms, it remains unknown if these patterns relate to cognitive and executive functional deficits characteristic of the schizophrenia syndrome (26). It may be possible that alterations in thalamo-cortical function (especially the PFC component) in part relate to cognitive deficits observed in this illness.

Additional questions still exist pertaining to the cross-diagnostic relevance of these observations. As noted above, it was shown by Anticevic and colleagues that qualitatively similar patterns of thalamic dysconnectivity are apparent in bipolar illness (although smaller in magnitude than those found in schizophrenia). It remains to be determined if those bipolar patients with a history of co-occurring psychosis are quantitatively more similar to alterations identified in schizophrenia (123). Such finer-grained cross-diagnostic investigations have further potential to inform and refine the clinical relevance of the identified marker. These studies should be complemented with targeted efforts to improve the classification provided by Anticevic and colleagues and allow for even more precision in harnessing this putative biomarker. Recently Fox and Greicius discussed progress in neuropsychiatric studies using resting-state connectivity. They appropriately concluded at the time that in schizophrenia there has been remarkably little progress in producing replicable results (34), perhaps owing to the complexity and heterogeneity of this neuropsychiatric illness noted above. These recent studies reviewed here, which collectively focused on identifying patterns of thalamo-cortical disruption in schizophrenia, may be converging on a parsimonious final common pathway of this complex disease, at least at the neural systems level (43). In that sense, these effects may be one of the better-replicated findings in the schizophrenia connectivity literature to date, offering promise for biomarker development and refinement.

A longer-term goal will entail bridging this neural system-level marker of schizophrenia with evolving cellular-level hypotheses of schizophrenia neuropathology. It remains unknown how the identified neural system-level markers relate to hypotheses that propose disruptions at the cellular level in schizophrenia. For instance, Anticevic and colleagues articulate a possible role of the disruption in cortical excitation (E) and inhibition (I) balance within the cortical microcircuit in producing system-wide disruptions, which may occur in schizophrenia (see next section) and in turn affect cognition (162). It remains unknown, however, how such alterations can scale to produce the presently observed pattern of aberrant thalamo-cortical connectivity. A corollary of this hypothesis relates to observations in bipolar illness, which was associated with an intermediate pattern of thalamo-cortical alterations. In bipolar illness different cellular-level hypotheses have been proposed from those hypothesized to occur in schizophrenia (112, 163, 164) [although some authors have articulated shared disturbances in GABA interneuron function (165)]. Therefore, either distinct mechanisms operate in these different neuropsychiatric conditions that converge on the same alterations or there may be, at least in part, a shared alteration in some of the same mechanisms across the two conditions. That is, it is possible that some patients with bipolar illness share some of the features of cellular neuropathology that affect patients with schizophrenia (165), especially those bipolar patients who present with co-occurring psychosis (166). Moreover, alterations along a number of distinct neurotransmitter pathways, involving a confluence of glutamate (7, 167), GABA (19), and dopamine disturbance (106), could jointly converge on a profound disturbance in thalamo-cortical function. In the upcoming sections, we discuss additional neuroscientific tools, namely pharmacological neuroimaging and computational modeling, which can be combined to help elucidate the role of specific cellular and synaptic mechanisms in observed system-level disruptions that occur in schizophrenia.

## Pharmacological Neuroimaging – Toward a Mechanistic Understanding of System-Level Disruptions in Psychiatric Illness

We presented evidence, supported by several emerging studies, for profound alterations in thalamo-cortical information flow in schizophrenia, as well as evidence for alterations in PFC connectivity. Yet, the mechanisms that could inform rationally guided pharmacotherapy for these disturbances in schizophrenia remain unknown. One leading mechanistic hypothesis proposes possible disruptions in the E/I balance in the cortical micro-circuitry resulting from hypo-function of the NMDAR (7), which might affect cortical computations, leading to large-scale dysconnectivity (14). A way to link such pharmacological mechanisms to neural system-level observations is to directly compare clinical patient studies and results following pharmacological manipulations, or to separately test the effects of pharmacological manipulations in healthy volunteers.

A powerful candidate approach is to use ketamine, a non-competitive NMDAR antagonist and a leading schizophrenia pharmacological model, which transiently, reversibly, and safely induces characteristic schizophrenia symptoms in healthy volunteers (7). Here pharmacological manipulations provide a method with which researchers can test the effects of a given neurotransmitter perturbation in a constrained, causal, and hypothesis-driven way. Moreover, such synaptic hypotheses can be implemented directly into computational models to generate experimental predictions (discussed in the last section). A prevailing hypothesis regarding ketamine’s effects on cortical micro-circuitry proposes preferential antagonism of interneurons with subsequent disinhibition of pyramidal cells (168), a mechanism we implemented in a recent computational modeling investigation (58) (see below for a discussion). As an extension of this hypothesis, a cortex-wide disruption in E/I balance might de-stabilize thalamo-cortical information flow in ways observed in schizophrenia (or other neuropsychiatric conditions). It should be noted that it still remains unclear what the relative contribution is of NMDARs on pyramidal cells (169) versus interneurons (58) may be in relation to the hypothesized alterations in E/I balance. Understanding the relative contribution of such cell-specific receptor alterations remains an important future direction, which could inform targeted pharmacotherapies. Such detailed studies of how cellular-level alterations could give rise to thalamo-cortical alterations following pharmacological manipulations remain to be done. Nevertheless, there is emerging evidence from a few focused investigations detailing the effects of ketamine on large-scale cortical connectivity. These investigations provide preliminary clues for how ketamine’s effects on large-scale systems may resemble effects seen in schizophrenia.

**Figure 4:**
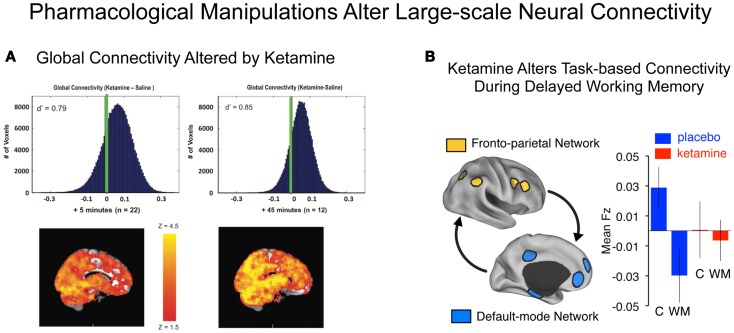
**Characterizing connectivity alterations using pharmacological neuroimaging**. While functional connectivity neuroimaging alone has been a powerful tool for characterizing neural system-level alterations in schizophrenia, it is ultimately a correlational tool. That is, we are examining alterations in the associations as a function of illness presence or absence. To move toward understanding the possible role of specific neurotransmitter mechanisms in schizophrenia, however, neuroimaging studies can be combined with pharmacological manipulations (47, 175). Such causal experimental manipulations can shed light on the role of specific neurotransmitter systems in schizophrenia (15). **(A)** Driesen and colleagues have shown that administration of ketamine profoundly altered the global connectivity of the brain. Specifically they demonstrated that the GBC measure increased following ketamine administration, possibly reflecting a hyper-glutamatergic state or a state of cortical disinhibition (54) consistent with animal models (111, 176). Formal computational models are needed in order to provide a deeper intuition for such pharmacological effects on large-scale neural systems. **(B)** Anticevic and colleagues have shown that administration of an NMDAR antagonist, ketamine, alters the connectivity of large-scale anti-correlated neural systems during performance of a working memory task (18). Although this connectivity study was performed during task performance rather than rest, it illustrates a proof of concept for how a pharmacological challenge can alter connectivity in a causal way. Note: figures adapted with permission from Driesen and colleagues (54) and Anticevic and colleagues (18).

For instance, a recent resting-state connectivity study by Driesen and colleagues investigated the effects of acute ketamine administration to healthy volunteers on large-scale global cortical connectivity (Figure 4A). The authors use a resting-state connectivity approach similar to that applied by Cole and colleagues in chronic schizophrenia (40), extended to include the entire brain (i.e., all voxels without imposing a PFC restriction). A number of studies have demonstrated that NMDAR antagonist administration is associated with excessive pyramidal cell activity, increases extracellular glutamate levels (16), and increases in perfusion and cortical metabolism (48–51, 53). A logical extension of this hypothesis is that administration of ketamine may profoundly affect large-scale cortical connectivity. Consistent with pre-clinical studies, Driesen and colleagues found that NMDAR antagonist administration resulted in a global elevation of functional connectivity (i.e., everywhere in the brain). This observation is broadly consistent with cellular-level hypotheses of ketamine’s effects on glutamate release, which may increase coupling of cortical circuits at rest by increasing the E/I ratio (i.e., decreasing cortical microcircuit inhibition). It is, however, critical to point out that chronic schizophrenia has typically been associated with reductions in cortical connectivity (170) and activation (70), especially in the PFC (11), as described above. Therefore, there are at least some evident discrepancies between the effects of pharmacological models such as ketamine and the actual illness (54). This important discrepancy between ketamine’s effects on PFC circuits and observations in schizophrenia should be reconciled in prospective studies that directly compare pharmacological and clinical effects using resting-state connectivity measures. One possible factor that could explain differences between NMDAR antagonist effects and chronic schizophrenia is that such pharmacological models may be relevant only to specific patient subgroups or illness stages. One possibility is that the increased connectivity under ketamine is similar to the early stages of psychotic illness. This hypothesis is consistent with elevated glutamate levels reported early in the illness course (113, 171). It is also consistent with the observation that ketamine tends to produce symptoms associated with incipient illness stages, rather than auditory hallucinations that occur in frank psychosis and chronic schizophrenia (15, 49, 172–174). Moreover, significant functional dynamical changes may occur during schizophrenia progression (46) that could profoundly affect PFC function, structure, and integrity. This hypothesis is supported by recent meta-analytic findings reporting decreases in glutamate across the illness progression (113). Whether such alterations across the schizophrenia illness course are reflected in PFC connectivity changes remains unknown, as does ketamine’s impact on PFC functional network architecture. Future pharmacological studies as well as cross-sectional and longitudinal clinical investigation are needed to test this hypothesis. In addition, careful pre-clinical experiments could possibly inform such hypotheses (118).

A complementary area of research has investigated the effects of ketamine on functional connectivity (177) in relation to its potential anti-depressant effects (178). While a comprehensive treatment of anti-depressant effects of ketamine is beyond the scope of this review, it is important to note that studies examining its effects on glutamateric pathways in the context of mood symptoms (178) may be highly informative for developing our understanding of its relevance to schizophrenia (111). Briefly, emerging models in this area postulate that ketamine may act as anti-depressant by promoting synaptic plasticity via intra-cellular signaling pathways, ultimately promoting brain-derived neurotrophic factor expression via synaptic potentiation (179) and in turns synaptic growth (178). In that sense, acute NMDAR antagonism may promote synaptic plasticity along specific pathways impacted in mood disorders, such as ventral medial PFC (180, 181, p. 916). Conversely, when administered to patients diagnosed with schizophrenia, NMDAR antagonists seem to worsen their symptom profile (182), perhaps by “pushing” an already aberrantly elevated glutamatergic signaling profile upward. Collectively such dissociable effects of ketamine may imply that along distinct circuits there may be an inverted-U relationship between ketamine’s effects and symptoms: depressed patients may be positioned on the low end of the inverted-U (178) and schizophrenia patents may be positioned on the higher end (183). Both task-based and resting-state functional connectivity techniques are well positioned to interrogate such system-level effects of NMDAR antagonists in humans.

As an example of such an approach, another study examining the effects of ketamine by Anticevic and colleagues focused on understanding the functional impact of NMDAR antagonism on the organization of the large-scale, anti-correlated neural systems (Figure 4B). This pharmacological neuroimaging investigation was explicitly focused on understanding ketamine effects on WM. However, in the context of this cognitive question, Anticevic and colleagues also assessed whether the task-based functional connectivity of large-scale neural systems is affected by ketamine administration. As we noted above, such task-based connectivity approaches can be useful to pinpoint how large-scale systems are affected during specific cognitive operations. The authors found that an acute administration of a low ketamine dose profoundly altered the typically observed anti-correlated structure of the large-scale neural systems – namely the task-positive fronto-parietal regions (184) and the task-negative regions (typically termed the default-mode network, DMN) (28). In line with this observed decrease in functional connectivity, Driesen and colleagues (185) also found a reduction in WM task-based functional connectivity along fronto-parietal areas (when using a DLPFC seed) following ketamine administration. The investigators reported *increased* functional connectivity of the DLPFC during rest using the exact same seed. This set of observations in particular sheds light on how a pharmacological manipulation such as NMDAR antagonism may have profoundly different effects in the context of a cognitive task (e.g., WM) and during rest. The mechanisms behind this observed difference are beyond the scope of this review and will be discussed in forthcoming studies.

Briefly, it may be possible that large-scale, network-level synchrony during WM is critically dependent on appropriate content-specific signals between neural subpopulations (186). In contrast, the BOLD fluctuations during rest likely relate to coupling between regions at the infra-slow-frequency ranges (187). It may be possible that a reduction in appropriate task-evoked synchrony by NMDAR antagonism reduces the ability of a network to form a coherent and optimal level of functional connectivity during WM. This may be exacerbated by an amplification of shared “noise” in the system, which is reflected in the apparent hyper-synchrony at rest (54). Collectively, therefore, an NMDAR antagonist may “disinhibit” the system, which gives rise to infra-slow, spatially distributed fluctuations across large areas of cortex that manifest in aberrant hyper-connectivity at rest. Precisely due to this elevated background noise, in combination with disrupted capacity for appropriate task-evoked synchrony, the net effect during WM may be a reduction in functional connectivity (which contrasts with observations at rest). Moreover, task-evoked activity can suppress the slow fluctuations associated with rest (188); if this suppression were weakened in schizophrenia, the “signal-to-noise” ratio would be degraded and task-based functional connectivity could be reduced. Because task-based and resting-state synchrony may be separated by timescale, EEG studies, combined with fMRI, could potentially address such hypotheses (189–191). Moreover, precisely because of such important differences between task and rest in certain contexts (e.g., ketamine manipulation) it remains important for task-based and resting-state investigations to inform one another. In the upcoming section, we explicitly discuss the recent developments in computational modeling that can provide a platform for formal integration of neuroscience theory both in the context of resting-state studies as well as formal task-based experiments.

## Bridging Levels of Analysis via Computational Modeling

Above we discussed recent clinical and pharmacological neuroimaging findings that shed light on the nature of large-scale neural system alterations in schizophrenia. In particular, the pharmacological experiments provide a causal method to explicitly manipulate specific neurotransmitter mechanisms that may be involved in schizophrenia. In that sense, these studies can begin to address given neurotransmitter contributions to neural system-level and behavioral alterations observed in schizophrenia. Still, these studies cannot measure synaptic and cellular-level phenomena alone. Therefore, one possible methodological integration involves a formal link between such pharmacological/clinical experiments and computational models that contain this level of functional detail. One branch of computational model that provides a particularly productive platform involves biophysically based models that contain the relevant synaptic mechanisms (57, 192) thought to be disrupted in neuropsychiatric illness (55). Such microcircuit models have been already harnessed to make predictions in the context of ketamine experimental manipulations of WM (58) (discussed more below). There is, however, an ongoing need to scale such models to the level of neural systems to provide relevant predictions for both resting-state and task-based clinical and pharmacological studies.

**Figure 5:**
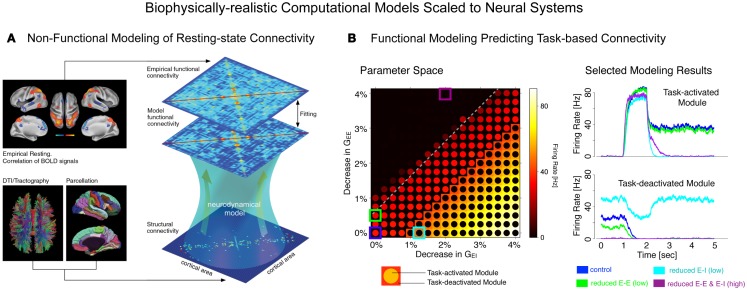
**Computational modeling of system-level effects via biophysically realistic computational approaches**. While detailed microcircuit models have made an impact on our understanding of cortical dynamics (56), the challenge remains to scale such models to incorporate dynamic interactions across large-scale neural systems, which are likely profoundly affected in schizophrenia (and other severe neuropsychiatric conditions). **(A)** A recently published elegant study by Deco and colleagues (59) illustrates an approach where a biophysically realistic model of cortical computations has been applied to understand the generation of slow-frequency fluctuations in the BOLD signal. The authors used diffusion-spectrum imaging to anatomically constrain the model and in turn fitted the modeling results to empirically-derived resting-state functional connectivity data. The result illustrates that coherent fluctuations in the BOLD signal (i.e., resting functional connectivity) may emerge from a system that is at the edge of chaos, allowing linear but transient departures in neuronal firing rates. **(B)** Anticevic and colleagues have used a functional model of working memory, a cognitive process that is profoundly affected in schizophrenia (26), to better understand the role of NMDA receptor function in the interaction of large-scale anti-correlated neural systems. Specifically they studied the functional antagonism present during a cognitive task between the task-activated (fronto-parietal module) and task-deactivated (default-mode module) networks (196). Following a complete parameter sweep (left), the authors found that a small perturbation of the NMDARs on inhibitory interneurons within each cortical microcircuit captured the firing that was observed experimentally following ketamine administration in healthy volunteers (18). Collectively, these studies offer examples for how biologically constrained modeling approaches can be applied to understand large-scale neural system physiology in both resting-state (non-functional) and task-based (functional) settings. Note: **(A)** of the figure was adapted with permission from Deco and colleagues (59).

Recent computational models have been developed to explicitly capture how the global pattern of resting-state functional connectivity arises through cortico-cortical interactions (193). In particular, modeling studies in this area have focused on the extent to which functional connectivity can be predicted by long-range anatomical connectivity (Figure 5A). The dynamic interactions between neural populations will also shape functional connectivity. The starting point for these models is an anatomical coupling matrix reflecting long-range connections between cortical regions, derived either from tracer studies in macaque monkeys or from diffusion tractography in humans. The activity of a local region (a node in the large-scale network) follows some dynamics and is shaped by input from other areas, propagating via the long-range connections. Ongoing activity, either due to chaos or noise, produces fluctuations in the activity across the network. The functional connectivity of the model can then be calculated and compared to functional connectivity observed in experiments. Honey and colleagues (194) studied long-range chaotic synchronization when local nodes follow oscillatory dynamics, using connectivity from human diffusion tractography. They found that the global dynamics of the network could partially explain the presence of strong functional connections between regions that lack direct anatomical connection. Cabral and colleagues (195) used a similar oscillatory model with connectivity data from healthy subjects, and parametrically varied the overall strength of long-range connections. They found that decreasing long-range connection strength altered functional connectivity patterns in a manner similar to those observed in schizophrenia (9), with reduced overall functional connectivity strength and changes in certain graph-theoretic measures of the functional connectivity matrix.

Deco and colleagues (59, 193) extended this approach, deriving long-range connectivity from human diffusion tractography and implementing the local node dynamics with a biophysically based model of a cortical microcircuit. In particular, the local microcircuit incorporates recurrent excitation with realistic synaptic dynamics, and the strength of recurrent excitation enables multi-stable dynamics that can subserve cognitive computations such as WM (197, 198). Noisy background inputs to nodes induce fluctuations in activity that are shaped into correlated patterns by long-range coupling between nodes through the anatomically derived connectivity. Functional connectivity in the model is given by the pattern of these correlated fluctuations. The authors parametrically varied two global parameters with biophysical relevance: the strength of recurrent excitation within local nodes, and the strength of long-range connections between nodes. The strengths of local and long-range connections combine to provide recurrent excitation in the network. They found that the similarity between model and experimental functional connectivity patterns was maximized when the network’s baseline state is near the boundary in parameter space between stability and instability induced by excess excitation. These studies reveal that the pattern of functional connectivity in the model is sensitive to both the pattern of anatomical connectivity and the physiological parameters that scale local and long-range connections. The biophysical basis of these models makes them directly applicable to address the dynamical consequences of anatomical and physiological changes induced by disease processes or pharmacological manipulation. Changes in the strengths of local and long-range connections may induce differential effects in the patterns of functional connectivity. Therefore, fitting models to functionally connectivity in patients could distinguish among distinct synaptic alterations. This approach could also potentially reveal the effects of complex drug actions on local circuit tuning and long-range interactions.

The models described above were explicitly constructed to simulate resting-state fluctuations, rather than to implement a particular function such as WM. Nevertheless, functional models can still make predictions that can be tested using functional connectivity, especially task-based functional connectivity (81). To this end, Anticevic and colleagues extended a microcircuit model of WM to study interactions between large-scale networks and their disruption by synaptic perturbation (Figure 5B) (18). The biophysically based model consists of two modules of spiking microcircuits: one that is task-activated and capable of WM computations, and one that is task-deactivated from a high-activity baseline state (hypothesized to model the activity pattern of the default-mode network in the context of cognitive activation). The interactions between these modules are mutually suppressive, via long-range projections onto inhibitory interneurons, a feature founded on findings of anti-correlated fluctuations between task-positive and default-mode networks (30). Within the model authors specifically studied the functional impact of disinhibition via NMDA hypo-function on interneurons, to relate such microcircuit hypotheses to neural changes observed under ketamine administration. The authors found that disinhibition of the entire network results in a failure to shut off the default-mode module, impairing the pattern of activation and deactivation during WM tasks. This modeling study provides one example of how a microcircuit model of a specific cognitive process (i.e., WM) can be scaled to incorporate system-level interactions and make predictions relevant for task-based functional connectivity. In addition, one strength of computational models is the ability to systematically explore different operating regimes in the space of model parameters. For example, Anticevic and colleagues contrasted reductions in recurrent excitation onto interneurons versus pyramidal cells, generating testable predictions for elevated versus reduced excitation/inhibition balance. As a test of the model architecture and operation, the authors analyzed task-based functional connectivity between task-activated and task-deactivated networks, computed across trials during the delay period of a WM task. Under control conditions, the two networks exhibited robust negative correlation, in line with effective antagonistic interactions in the model. In contrast, under ketamine administration this negative correlation disappears (Figure 4B), in line with the model prediction that the task-positive module cannot effectively suppress the hyperactive default-mode module. Collectively, this study provides preliminary evidence that functional models can be directly related to functional connectivity predictions in the context of WM. Future computational/experimental studies should be designed to extend this framework to more complex processes and symptoms that may be disrupted in schizophrenia. We argue that such ongoing efforts for the integration of theory, pharmacological experiments and clinical work will be a vital path for the field of clinical neuroscience to provide testable and rationally guided advances for understanding disease mechanisms and putative treatments.

## Concluding Remarks

Collectively, we articulated recent focused developments in three areas of clinical neuroscience of schizophrenia: (i) we reviewed methodological advances in resting-state functional connectivity that were directly translated to understand neural system-level disturbances in schizophrenia. We specifically focused on data-driven techniques that offer a promising way to detect disrupted connectivity while bypassing the likely complexity and regional heterogeneity of network alterations that are present in schizophrenia. We also discussed ongoing developments in studies of thalamo-cortical dysconnectivity in schizophrenia that are directly informed by influential theoretical models of the illness. (ii) We highlighted select pharmacological studies of the NMDARs that offer a causal way to understand neural system alterations in psychiatric illness. (iii) We articulated the developments in biophysically based computational modeling studies that provide a platform for testing specific synaptic alterations. In turn, we demonstrate that such models can potentially be scaled to the level of neural systems to make relevant predictions for both resting-state or task-based connectivity experiments. We argue that the ongoing blend of these three approaches can provide advances in the field of clinical neuroscience that create a final output that is much greater than the sum of its parts.

## Conflict of Interest Statement

John H. Krystal consults for several pharmaceutical and biotechnology companies with compensation less than $10,000 per year. All other authors declare that they have no conflict of interest.
